# Myocarditis in Patients With Antisynthetase Syndrome: Prevalence, Presentation, and Outcomes

**DOI:** 10.1097/MD.0000000000000798

**Published:** 2015-07-02

**Authors:** Céline Dieval, Christophe Deligny, Alain Meyer, Philippe Cluzel, Nicolas Champtiaux, Guillaume Lefevre, David Saadoun, Jean Sibilia, Jean-Luc Pellegrin, Eric Hachulla, Olivier Benveniste, Baptiste Hervier

**Affiliations:** From the Service de Maladies Infectieuses et Maladies du Sang, Centre Hospitalier de Rochefort (CD); Service de Médecine Interne, Centre Hospitalier Universitaire de Fort de France (CD); Service de Rhumatologie, Centre National de Référence des Maladies Autoimmunes et Systémiques Rares, Hôpitaux Universitaires de Strasbourg (AM, JS); Service de Radiologie Vasculaire et Interventionnelle (PC); Département de Médecine Interne et d’Immunologie Clinique, Centre national de Référence des Maladies Neuromusculaires, DHU I2B, Hôpital Pitié-Salpêtrière, 47-83 Boulevard de l’Hôpital, Paris (NC, DS, OB, BH); Service de Médecine Interne – Centre national de Référence des Maladies Autoimmunes et Systémiques Rares, Hôpital Claude Huriez, Université de Lille, Lille (GL, EH); and Service de Médecine Interne, Hôpital Haut-Lévêque, Pessac, France (J-LP).

## Abstract

Antisynthetase syndrome (aSS) corresponds to an overlapping inflammatory myopathy identified by various myositis-specific autoantibodies (directed against tRNA-synthetases). Myocardial involvement in this condition is poorly described.

From a registry of 352 aSS patients, 12 cases of myocarditis were retrospectively identified on the basis of an unexplained increase in troponin T/I levels associated with either suggestive cardiac magnetic resonance imaging (MRI) findings, nonsignificant coronary artery abnormalities or positive endomyocardial biopsy.

The prevalence of myocarditis in aSS is 3.4% and was not linked to any autoantibody specificity: anti-Jo1 (n = 8), anti-PL7 (n = 3), and anti-PL12 (n = 1). Myocarditis was a part of the first aSS manifestations in 42% of the cases and was asymptomatic (n = 2) or revealed by an acute (n = 4) or a subacute (n = 6) cardiac failure. It should be noted that myocarditis was always associated with an active myositis. When performed (n = 11), cardiac MRI revealed a late hypersignal in the T1-images in 73% of the cases (n = 8). Half of the patients required intensive care. Ten patients (83%) received dedicated cardiotropic drugs. Steroids and at least 1 immunosuppressive drug were given in all cases. After a median follow-up of 11 months (range 0–84) 9 (75%) patients recovered whereas 3 (25%) developed a chronic cardiac insufficiency. No patient died.

The prevalence of myocarditis in aSS is similar to that of other inflammatory myopathies. Although the prognosis is relatively good, myocarditis is a severe condition and should be carefully considered as a possible manifestation in active aSS patients.

## INTRODUCTION

Antisynthetase syndrome (aSS) belongs to the category of autoimmune inflammatory myopathies and is characterized by the presence of various but mutually exclusive anti-tRNA-synthetase autoantibodies. Its spectrum often includes myositis with interstitial pneumonia, Raynaud's phenomenon, inflammatory polyarthralgia/polyarthritis, and mechanic's hands.^1,2^ Cardiac involvement in inflammatory myopathies is polymorphic and includes subclinical (electromyographic) changes or different features like congestive heart failure,^3,4^ pulmonary hypertension,^5^ pericarditis,^6^ and myocarditis. This latter manifestation seems to be particularly rare in aSS, with only a few case reports published to date.^7,8^ Although potentially severe, this condition could be underestimated.

Myocarditis can occur in contexts other than myopathies, including infectious or inflammatory diseases or hypereosinophilia.^7,8^ Diagnosing myocarditis remains difficult and the clinical classification criteria are still a matter of debate. Based on the WHO classification, myocarditis is an inflammatory myocardial injury and is defined based on an endomyocardial biopsy (EB) with specific histological and immunological features.^9–11^ However, performing an EB is risky and is now only rarely performed, especially when cardiac-magnetic resonance imaging (MRI) is suggestive.^9–13^

Diagnosing myocarditis remains a challenge in patients with an autoimmune disease, particularly with inflammatory myopathies. This is because the clinical manifestations can be subclinical, nonspecific, or concealed by other clinical features, including pericarditis or lung involvement. It is however crucial to diagnose myocarditis, due to the need for specific treatments, and to avoid a potentially fatal early evolution or the development of chronic heart failure at a later stage.

We have conducted the largest retrospective series to date, with the objective of describing myocarditis in the context of aSS, specifying its prevalence and outcomes.

## PATIENTS AND METHODS

The myocarditis cases occurring during aSS (n = 12) were retrospectively selected from the French national registry (n = 352, 10 university centers, 2000–2014). Myocarditis was defined by the occurrence of acute (<24 hours) or subacute (24–72 hours) cardiac symptoms associated with either increased troponin I or T (which are highly specific for cardiomyocyte necrosis)^14,15^ and suggestive cardiac-MRI or EB (patient # 10) and in the absence of any other significant cause (including viral infection, coronaropathy, or valvulopathy). It should be noted that 2 eligible patients who died before specific morphological examination were not included, despite a compatible myocarditis diagnosis. Demographic data, clinical, biological and morphological findings, treatments, and outcomes were retrospectively reviewed by CD,^1^ CD,^2^ and BH, using the same form. The study was approved by local ethical committees, and patients were reported anonymously, in accordance with French law.

## RESULTS

Of the entire cohort, 12 patients (3.4%, woman/man ratio = 3, median age 54, range 17–67) were identified as meeting the classification criteria. Cardiovascular risk factors were found in half of the cases (n = 6) but rarely significant. Their distribution was smoking habit (n = 2), systemic hypertension (n = 2), dyslipidemia (n = 2), obesity (n = 2), and mild diabetes mellitus (n = 3), see Table 1 .

**TABLE 1:**
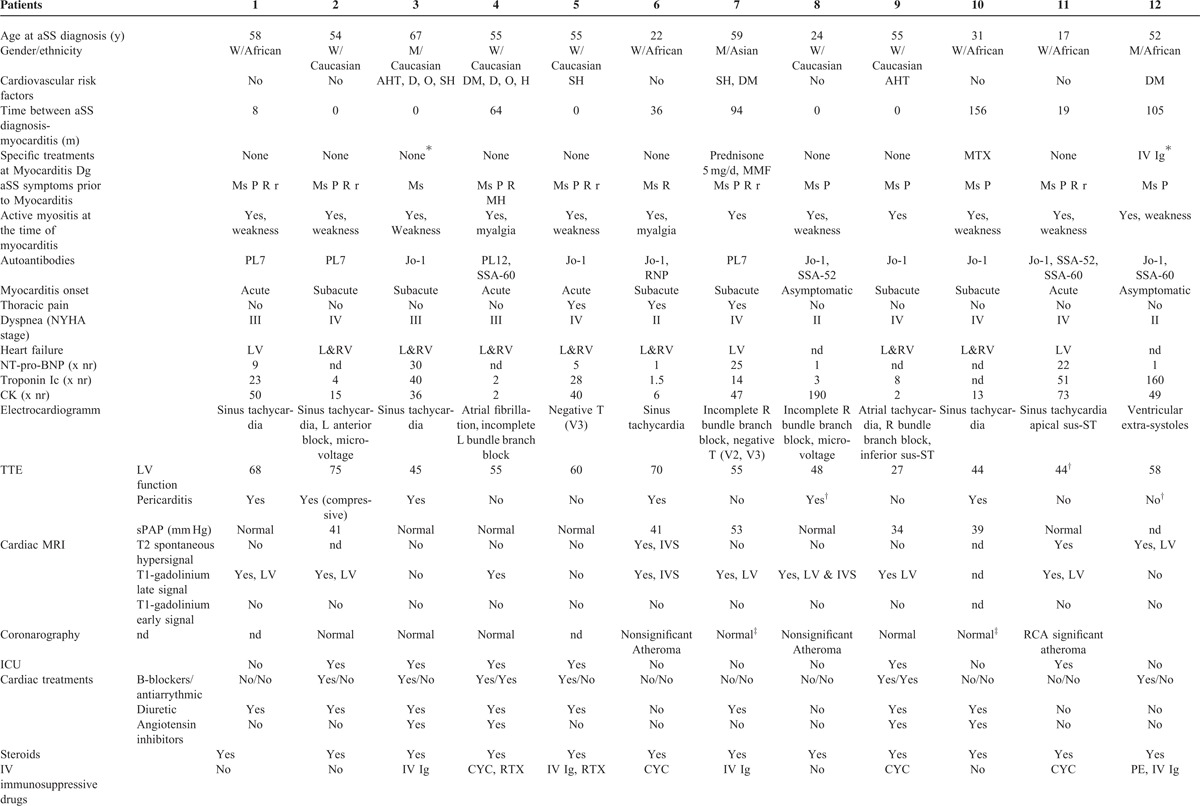
Patient Characteristics

**TABLE 1 (Continued):**
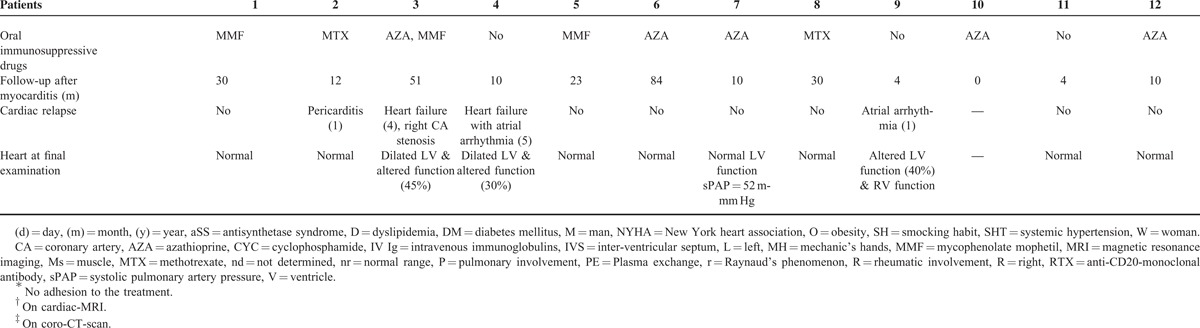
Patient Characteristics

Among the clinical features of aSS, myocarditis was one of the first aSS manifestations in 42% (n = 5) of the cases. In the other cases, myocarditis occurred during the follow-up period (median 36 months, range 8–156). In only 3 (43%) of these cases were the patients treated with immunosuppressive drugs at the time of the myocarditis diagnosis. Myocarditis manifestations included left and/or right ventricular dysfunction (n = 10), which was often severe (leading to congestive heart failure with New York heart association stage ≥III: n = 9). Intensive care was indeed required 6 times (50%). Chest pain was reported 3 times (25%). These clinical features were acute (n = 4) or subacute (n = 6). Two patients presented without any symptom consistent with heart failure. Electrocardiography showed sinusal tachycardia (n = 6), heart branch block (n = 5), and/or atrial arrhythmias (n = 2). Repolarization defects were also observed (n = 4, see Table 1 ).

Troponin I or T levels were significantly elevated in all of the tested cases (19 times the normal range, 1.5–160), as was creatine kinase (38 times the normal range, 2–190).

Transthoracic echocardiography revealed pericarditis in 6 cases (50%), with compression in 1 case. Left-ventricular ejection fraction was decreased in 7 cases (median of 46%, 27–55) and normal in the remaining 5 cases. Two patients had a significantly dilated ventricle but none was hypertrophic. No significant valvulopathy was observed and pulmonary hypertension was at least “possible,”^16^ in 4 cases (33%).

**FIGURE 1:**
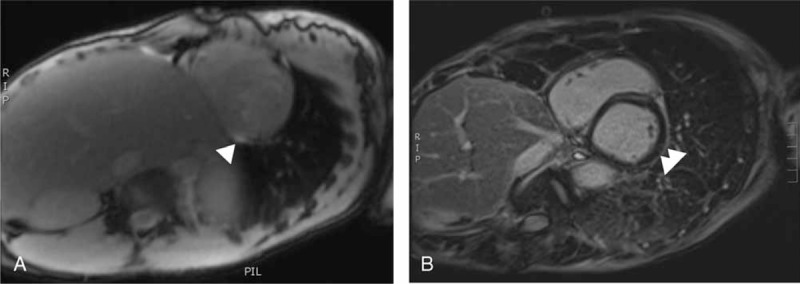
Example of the cardiac-MRI features suggestive of myocarditis. (A) White arrow: myocardium spontaneous T2-hypersignal of the left ventricle consistent an inflammatory process (edema, patient 11). No pericardial effusion. (B) White arrow: myocardium late-T1-post-gadolinium enhancement of the left ventricle consistent an inflammatory process. No pericardial effusion (patient 7).

Cardiac-MRI (n = 11) revealed a spontaneous T2 hypersignal in 3 cases (27%) and/or a T1-gadolinium late signal suggestive of myocarditis in 7 cases (73%, see Figure 1) and no T1-gadolinium early signal was observed. In the remaining case, EB showed focal active and chronic inflammatory lesions with both lymphocytes and neutrophils invading the myocardium, consistent with the diagnosis of myocarditis. Coronarography (n = 7) and coro-CT-scan (n = 2) were normal or did not show significant atheroma explaining the ventricular lesions. Viral screening was negative (n = 11), with the exception of patient 7, who presented with a slightly positive cytomegalovirus blood replication (blood copy number by polymerase chain reaction = 3.2 log). Causality was however not confirmed in this case.

According to these findings, and based on Sagar et al,^10^ myocarditis was ruled a definite myocarditis in 1 case with EB (8%), a probable acute myocarditis in 6 cases (50%), and only a possible subclinical acute myocarditis in the 5 remaining cases (42%).

Aside from heart involvement, other clinical aSS features observed at the time of aSS initial diagnosis or during the follow-up period were similar to those commonly described in larger aSS cohorts.^1,2^ These manifestations however included active myositis in all cases, interstitial pneumonia in 83% of the cases, and less commonly dermatologic and rheumatic manifestations (see Table 1 ). Of note, no patient presented with erosive arthritis or aSS-rheumatoid arthritis overlapping syndrome. Various anti-tRNA-synthetase autoantibody specificities were found anti-Jo1 (n = 8), anti-PL7 (n = 3), and anti-PL12 (n = 1). The prevalence of other associated autoantibodies, including anti-Ro/SSA and anti-La/SSB, was similar to what has been reported previously.^2^ All patients were anticitrullinated peptide/protein antibody negative.

The treatments targeted both the heart and the immune system. Cardiac treatment consisted of diuretic (n = 9), beta-blockers (n = 6), angiotensin inhibitors (n = 4), antiarrhythmic drugs (n = 2), and inotropic drugs (n = 2). Pericarditis drainage was performed once. Immunosuppressive drugs initially included steroids in all of the cases associated with intravenous immunoglobulins (n = 4), intravenous cyclophosphamide (n = 4), and anti-CD20 monoclonal antibodies (n = 3). Oral immunosuppressive drugs were also administered, among which mycophenolate mophetil or azathioprine were the most common (n = 4 cases each, see Table 1 ). Of note, a second therapeutic line was necessary in 3 cases (30%).

With a median follow-up of 11 months (0–84), 2 different outcomes were observed. Eight patients (75%) presented with normal heart examinations, despite a relapse of pericarditis in 1 case. However, 3 patients developed chronic heart failure, with several relapsing episodes (n = 3). In these 3 cases, transthoracic echocardiography showed altered left ventricular function, with significant left ventricle dilation in 2 cases. No patient died.

Taken as a whole and consistent with Cooper et al,^9^ these results suggest the following myocarditis distribution: an acute myocardial infarction-like syndrome with normal coronary arteries (n = 6, 50%), heart failure with normal-sized or dilated left ventricle and hemodynamic compromise (n = 3, 25%), and heart failure with dilated left ventricle, new ventricular arrhythmia, and 3rd degree heart block (n = 3, 25%), respectively.

## DISCUSSION

This study showed a prevalence of myocarditis in aSS of 3.4%. However, the limitation of this study is inherent to its retrospective design, and these data could have been either underestimated due to severe undiagnosed cases^7^ or overestimated due to recruitment bias (all centers being tertiary care centers). There have been no reports to date in the literature providing this specific figure for aSS, but it is comparable to what has been reported for other inflammatory myopathies.^17^ Myocarditis was associated to pericarditis in half of the cases, which is more than expected, since the frequency of isolated pericarditis is roughly 10% in inflammatory myopathies.^3^ However, as confirmed by this study, pericarditis may be more frequent in the context of aSS.^6^

Myocarditis inaugurated aSS in 42% of the cases and was always associated with extra-cardiac symptoms in accordance with aSS activity, including active myositis in all cases. These data outline the importance of seeking extra-cardiac symptoms in cases of myocarditis to make the aSS diagnosis, which could be confirmed by the seropositivity of anti-tRNA-synthetase autoantibodies.

The extra-cardiac features were typical of aSS and did not predict the occurrence of myocarditis in this context. Clinical examination as well as first line biological and morphological exams is not very helpful in guiding the myocarditis diagnosis. In fact, dyspnea is common in aSS because of interstitial lung disease or respiratory muscle involvement. Electrocardiographic anomalies are nonspecific and have low sensitivity.^18^ Creatine kinase levels are difficult to interpret, particularly in the context of myositis, and troponin T and I are not a sensitive enough indicator for diagnosing myocarditis.^14,15^ Early echocardiography is absolutely necessary when diagnosing alternative diseases,^9,10^ as well as when evaluating the severity of myocarditis.^9,10^ However, no signs are specific to myocarditis on echocardiography. EB should therefore be the cornerstone of a positive diagnosis,^11^ but the patient's condition rarely allows for such an invasive procedure.

This leaves us with cardiac-MRI as the main noninvasive tool for the diagnosis of myocarditis. When performed early, T2-weighted images are suggestive of myocardic edema as a spontaneous hyperintensity. Early T1-contrast after the gadolinium injection reveals hypoperfusion zones, whereas late T1-post-gadolinium contrasts are consistent with inflammatory process and fibrosis.^9,10,12,13^ However, these delayed T1-post-gadolinium enhancements might not distinguish myocarditis from chronic scarring. It is generally accepted that the combination of these MRI features provides the highest sensitivity and specificity.^10^ However, all MRI features are not always concomitantly found and the mere presence of just one of these indicators is associated with a good positive predictive value (above 2/3).^10^ In this case series, T2-weighted was positive only 3 times (25%). This is likely due to the fact that the cardiac MRIs were performed rather late (up to 3 months after first cardiac symptoms, patient 3) and/or after the patients had begun a steroids treatment.

Since myocarditis implies severe organ involvement, it is an important condition to diagnose in aSS. Myocarditis can indeed lead to the development of dilated cardiomyopathy in 30% of cases.^19^ Moreover, in inflammatory myopathies, heart involvement seems to be a major cause of death (about 20% of the cases).^3,4,17,20^ In this series, though intensive care was required for half of the patients, no deaths occurred. Moreover and though 2 severe patients could have been missed, the patients survival does not differ from that of other aSS patients.^2^ Three patients (30%) developed chronic left ventricle dysfunction, among which 2 presented with dilated cardiomyopathy despite appropriate treatments. Thus, early and aggressive therapy, including immunosuppressive drugs, seems recommended.

In summary, although rare and difficult to diagnose, myocarditis must be carefully sought out in aSS patients, especially those with pericarditis. Starting specific treatments early on could be important to preventing the development of chronic dilated cardiomyopathy.

## KEY MESSAGES

Prevalence of aSS-related myocarditis is 3.4%, similar to that of other inflammatory myopathies. Although difficult in the context of aSS, the myocarditis diagnosis is important to make: the outcome can be severe and require specific medications.

## References

[1] Marguerie C Bunn CC Beynon HL Polymyositis, pulmonary fibrosis and autoantibodies to aminoacyl-tRNA synthetase enzymes. *Q J Med* 1990; 77:1019–1038. 2267280 10.1093/qjmed/77.1.1019

[2] Hervier B Devilliers H Stanciu R Hierarchical cluster and survival analyses of antisynthetase syndrome: phenotype and outcome are correlated with anti-tRNA synthetase antibody specificity. *Autoimmun Rev* 2012; 12:210–217. 22771754 10.1016/j.autrev.2012.06.006

[3] Lundberg IE The heart in dermatomyositis and polymyositis. *Rheumatology* 2006; 45:iv18–iv21. 16980718 10.1093/rheumatology/kel311

[4] Van Gelder H Charles-Schoeman C The heart in inflammatory myopathies. *Rheum Dis Clin North Am* 2014; 40:1–10. 24268006 10.1016/j.rdc.2013.10.002

[5] Hervier B Meyer A Dieval C Pulmonary hypertension in antisynthetase syndrome: prevalence, aetiology and survival. *Eur Respir J* 2013; 42:1271–1282. 23397301 10.1183/09031936.00156312

[6] Labirua-Iturburu A Selva-O’Callaghan A Vincze M Anti-PL-7 (anti-threonyl-tRNA synthetase) antisynthetase syndrome: clinical manifestations in a series of patients from a European multicenter study (EUMYONET) and review of the literature. *Medicine* 2012; 91:206–211. 22732951 10.1097/MD.0b013e318260977c

[7] Brady S Melath S Scalco RS Fatal cardiac involvement complicating antisynthetase syndrome. *BMJ Case Rep* 2014; 25: pii: bcr2014204409. 10.1136/bcr-2014-204409 PMC4154036 25155488

[8] Sharma K Orbai AM Desai D Brief report: antisynthetase syndrome-associated myocarditis. *J Card Fail* 2014; 20:939–945.pii: S1071-9164(14)00685-X. 25084215 10.1016/j.cardfail.2014.07.012 PMC4435564

[9] Cooper LT Jr Myocarditis. *N Engl J Med* 2009; 360:1526–1538. 19357408 10.1056/NEJMra0800028 PMC5814110

[10] Sagar S Liu PP Cooper LT Jr Myocarditis. *Lancet* 2012; 379:738–747. 22185868 10.1016/S0140-6736(11)60648-X PMC5814111

[11] Cooper LT Baughman KL Feldman AM The role of endomyocardial biopsy in the management of cardiovascular disease: a scientific statement from the American Heart Association, the American College of Cardiology, and the European Society of Cardiology. Endorsed by the Heart Failure Society of America and the Heart Failure Association of the European Society of Cardiology. *J Am Coll Cardiol* 2007; 50:1914–1931. 17980265 10.1016/j.jacc.2007.09.008

[12] Lurz P Eitel I Adam J Diagnostic performance of CMR imaging compared with EMB in patients with suspected myocarditis. *JACC Cardiovasc Imaging* 2012; 5:513–524. 22595159 10.1016/j.jcmg.2011.11.022

[13] Friedrich MG Sechtem U Schulz-Menger J Cardiovascular magnetic resonance in myocarditis: a JACC white paper. *J Am Coll Cardiol* 2009; 53:1475–1487. 19389557 10.1016/j.jacc.2009.02.007 PMC2743893

[14] Lauer B Niederau C Kühl U Cardiac troponin T in patients with clinically suspected myocarditis. *J Am Coll Cardiol* 1997; 30:1354–1359. 9350939 10.1016/s0735-1097(97)00317-3

[15] Smith SC Ladenson JH Mason JW Elevations of cardiac troponin I associated with myocarditis. Experimental and clinical correlates. *Circulation* 1997; 95:163–168. 8994432

[16] Galiè N Hoeper MM Humbert M Guidelines for the diagnosis and treatment of pulmonary hypertension. *Eur Respir J* 2009; 34:1219–1263. 19749199 10.1183/09031936.00139009

[17] Gupta R Wayangankar SA Targoff IN Clinical cardiac involvement in idiopathic inflammatory myopathies: a systematic review. *Int J Cardiol* 2011; 148:261–270. 20826015 10.1016/j.ijcard.2010.08.013

[18] Morgera T Di Lenarda A Dreas L Electrocardiography of myocarditis revisited: clinical and prognostic significance of electrocardiographic changes. *Am Heart J* 1992; 124:455–467. 1636589 10.1016/0002-8703(92)90613-z

[19] D’Ambrosio A Patti G Manzoli A The fate of acute myocarditis between spontaneous improvement and evolution to dilated cardiomyopathy: a review. *Heart* 2001; 85:499–504. 11302994 10.1136/heart.85.5.499 PMC1729727

[20] Zhang L Wang GC Ma L Cardiac involvement in adult polymyositis or dermatomyositis: a systematic review. *Clin Cardiol* 2012; 35:686–691. 22847365 10.1002/clc.22026 PMC6652370

